# Analysis of Behavior Trajectory Based on Deep Learning in Ammonia Environment for Fish

**DOI:** 10.3390/s20164425

**Published:** 2020-08-08

**Authors:** Wenkai Xu, Zhaohu Zhu, Fengli Ge, Zhongzhi Han, Juan Li

**Affiliations:** 1 School of Mechanical and Electrical Engineering, Qingdao Agricultural University, Qingdao 266109, China; 199401013@qau.edu.cn; 2 School of Science and Information, Qingdao Agricultural University, Qingdao 266109, China; hxydzz@qau.edu.cn (Z.Z.); hanzhongzhi@qau.edu.cn (Z.H.); 3 School of Management, Qingdao Agricultural University, Qingdao 266109, China; gfl@qau.edu.cn; 4 Key Laboratory of Security Control Technology for Complex System Driven By Big Data, Shandong University of Science and Technology, Qingdao 266590, China

**Keywords:** ammonia concentration, behavior analysis, deep learning, Faster R-CNN, fish, YOLO-V3

## Abstract

Ammonia can be produced by the respiration and excretion of fish during the farming process, which can affect the life of fish. In this paper, to research the behavior of fish under different ammonia concentration and make the corresponding judgment and early warning for the abnormal behavior of fish, the different ammonia environments are simulated by adding the ammonium chloride into the water. Different from the existing methods of directly artificial observation or artificial marking, this paper proposed a recognition and analysis of behavior trajectory approach based on deep learning. Firstly, the three-dimensional spatial trajectories of fish are drawn by three-dimensional reconstruction. Then, the influence of different concentrations of ammonia on fish is analyzed according to the behavior trajectory of fish in different concentrations of ammonia. The results of comparative experiments show that the movement of fish and vitality decrease significantly, and the fish often stagnates in the water of containing ammonium chloride. The proposed approach can provide a new idea for the behavior analysis of animal.

## 1. Introduction

With the development of society, the related research on aquaculture becomes more and more popular [1]. Fish, as the main crop in aquaculture, has become one of the high-value products in aquaculture. According to the statistics of Food and Agriculture Organization of the United Nations (FAO), the total amount of aquaculture in the world is 111,946,623 tons in 2017, in which the fish production is 53,402,661 tons. It can be seen that fish has been one of the main aquaculture objects, which accounts for about half of the total aquaculture. Therefore, how to improve the yield and efficiency of fish farming has become an important issue in fish farming research, and this is also an urgent problem to be solved which restricts the development of aquaculture industry. As we all know, the premise to solve this problem is to obtain the behavior analysis of fish through the behavior trajectory, find out the anomalies and laws to guide the breeding and prevent the occurrence of abnormal conditions.

In order to proceed the behavior analysis of fish, the recognition and location is necessary for the fish, then we can analyze the behavior of fishes. Currently, the existing methods for recognition and analysis can be classified into the qualitative methods and the quantitative methods. The qualitative methods include three kinds. The first kind of method is the complete manual method for recognition and analysis, such as the manual observation method or manual marking method [2]. The second kind of method is the fluorescent-based recognition and manual analysis method, which is using the fluorescent markers to locate the fish and manually analyze the behavior, such as [3]. The third kind of method is the video-based recognition and manual analysis method. In the third kind of method, a marker was put in the body of fish and the video was used to track the marker in the fishes’ bodies to detect the fish. This method still needs the manual behavior analysis for fish, such as [4]. The second and third kinds of methods are all invasive so that these methods all lead to the fish’s stress behavior, and the behavior analysis only can be done manually. The above methods are time-consuming and laborious, and the results of behavior analysis are susceptible to the subjective factors. Furthermore, only the qualitative results can be obtained, rather than the quantitative results and the behavior trajectory cannot be plotted.

In order to solve the problem that the manual analysis is influenced by the subjective factors and the quantitative results cannot be obtained, the quantitative methods are proposed to analyze the behavior of fish. The quantitative methods include two kinds. The first kind of method is based on appearance feature to recognize the objects and obtain the behavior trajectory, such as shape matching [5] and infrared reflection [6]. The second kind of method is based on image processing to recognize the objects and obtain the behavior trajectory [7], such as the inter-frame difference method [8] and optical flow method [9]. On this basis, some scholars combined the above methods with the other some methods for fish recognition, and proposed some improved methods. For example, [10] and [11] combined the inter-frame differences with the moving average background to optimize the traditional process of operation. However, these methods based on image processing are affected by the illumination, which results in the lower recognition accuracy when the illumination changes [12].

In conclusion, the most existing approaches of behavior trajectory analysis for fish need the manual participation and the analysis is only qualitative, so these approaches have some shortcomings such as strong subjectivity and low recognition accuracy. Few quantitative behavior trajectory analyses are only based on marking on the fish’s bodies, which has certain influence on fish’s behavior. This paper is inspired by artificial intelligence (e.g., [13,14,15]), so a deep learning-based behavior analysis approach is proposed, including the recognition and quantitative behavior trajectory plotting of fish with different ammonia concentration. However, to the best of our knowledge, we have not come across the research reports on the influence of environmental factors on the fish by plotting the behavior trajectory of fish, and we have not yet come across the reports of behavior analysis by using the deep learning and computer vision. Therefore, this paper takes ammonia environment as an example. Ammonia is highly toxic [16], and a small amount of ammonia can disturb the behavior of fish (e.g., [17,18,19]). This paper uses an approach based on deep learning to research the influence of environmental anomalies on the behavior of fish.

The contributions of this paper are as follows: (1) a new fish behavior analysis approach is proposed based on deep learning; (2) the results of the analysis are compared by using two kinds of usual deep learning approaches and the more suitable deep learning approaches is given to analyze the behavior; (3) the proposed approach provides a new train of thought for the behavior analysis and exploration of marine organism.

The rest of this paper is organized as follows. The used materials and methods are introduced in Section 2. The experimental results and the behavior are analyzed in Section 3. Some discussions are given on the results of experiment in Section 4. Conclusions are drawn in Section 5.

## 2. Materials and Methods

### 2.1. Experimental Materials and Device

In order to research the influence of ammonia on fish, ammonium chloride is added into water to imitate different concentrations of ammonia, which is produced by fish in intensive aquaculture environment. Three red goldfish of similar size are selected as the research objects. The trunk length of fish is about 6 cm and the trunk height is about 2 cm. Three experiments are done, and every experiment is conducted for two days. Three goldfish are selected, and every goldfish is used for one experiment. The fish is placed in a glass tank with 5.6 L of water, and the water is about 5 cm deep, which is about twice as much as the height of the fish body. The water level makes the swimming range of the fish remain roughly in the two-dimensional plane. On the first day, no ammonium chloride is added to the water to keep the normal environment. On the second day, the experimental conditions for every fish are the same as those of the first day except for changing the different concentrations of ammonium chloride. Every experiment is shot for two days and the shooting time is kept for 10 h between 8:30 and 18:30 every day. Before and after every shooting, the pH value and ammonia nitrogen content of water are detected at 8:25 and 18:35 in the fish tank.

**Figure 1:**
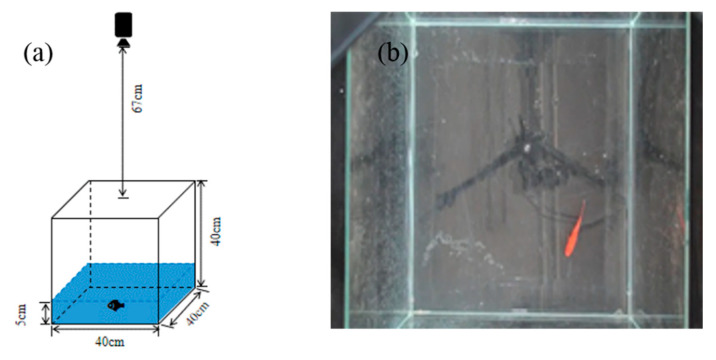
Experimental device. (**a**) schematic diagram of the experimental device, (**b**) actual shooting effect.

In order to reduce the influence of reflection on photography, a layer of black cloth is placed at the bottom of the fish tank. The size of fish tank is 40 cm × 40 cm× 40 cm. A Sony color camera is placed 67 cm high above the fish tank and it is fixed by a tripod. In order to observe the behavior of fish conveniently, 5.6 L of water is added to the tank. The water level is about 5 cm, and the camera distance is 102 cm away from the water surface. Figure 1 shows the schematic diagram of the experimental device and the effect of actual shooting. The used chemical reagents include ammonium chloride 3.92 g, a box of pH detector and a box of ammonia nitrogen detector. Videos and images are processed by MATLAB 2018a and Python 3.5 under Windows 10, and the process is accelerated by GPU.

### 2.2. Pre-Processing

In this paper, the format of the saved video is MPEG transport stream (MTS, a kind of video format) by the camera. In order to ensure the normal processing in MATLAB, MTS files are converted into the common audio video interleaved (AVI, a kind of video format) files through the video format conversion software “Format Factory”. By using the MATLAB programming, the 10 h video with AVI format is cut at the rate of one frame per second, and 36,000 images are obtained. As three experiments are carried out and every experiment is divided into two conditions, which are the normal environment and the abnormal environment with ammonia, a total of 21,600 images are collected. The format of the images is JPG.

Through the MATLAB programming, 1700 images are randomly cut from the 10 h video of the first experiment as the training and testing objects. The ratio of training set to the testing set is 7:3, that is to say, 1190 images are selected as training images and the left 510 images are selected as the testing images. We mark these images with the LABELIMG software under the Windows environment. The fish are similar in size and shape in the three experiments, so we use the marked data in the first experiment as the general data set for the three experiments.

### 2.3. Recognition and Location for Fish

#### 2.3.1. Recognition Based on Faster R-CNN

**Figure 2:**
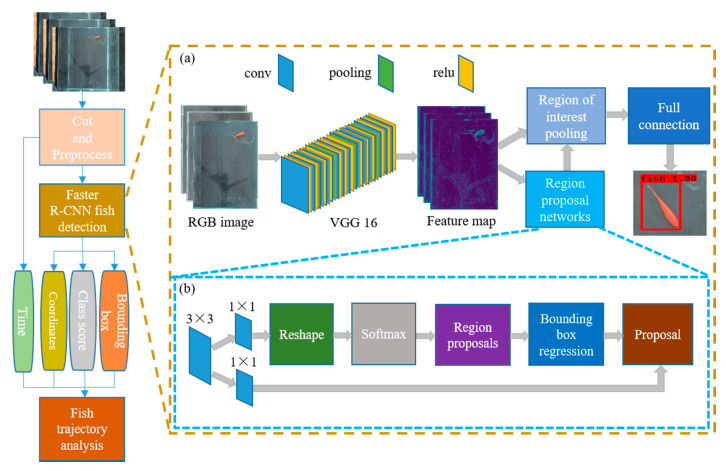
Recognition process of Faster region convolutional neural networks (R-CNN). (**a**) Structure of Faster R-CNN detector. (**b**) Detailed process of regional proposal networks (RPN).

In this section, the fish will be detected by using the Faster region convolutional neural networks (R-CNN) and the Figure 2 shows the recognition process based on Faster R-CNN. The cut and synthesized images are input to the Faster R-CNN and a series of parameters are obtained. These parameters can be processed to obtain the behavior trajectory of the fish. The Faster R-CNN is used to recognize and locate the object, so the Faster R-CNN detector will be introduced in the following part.

Figure 2a gives the structure of Faster R-CNN detector, which is composed of the following three parts:

##### Part I: Convolution

This layer is used to extract features of images for fish. The input is any size of the images, and the output is the feature map containing extracted features. The convolution is composed of convolution layers, rectified linear unit (ReLU) layers and pooling layers. In this paper, the Visual Geometry Group (VGG16) network is used. The VGG16 network can estimate images more accurately and occupy less space. The number 16 in VGG16 represents 13 convolutional layers and 3 full connection layers in the network. The section of convolution layers of VGG16 network contains 13 convolution layers, 13 ReLU layers and 4 Pooling layers. The detail function of every layer is as follows:

(1) Convolution layers: the purpose of the function is to achieve the parameter sharing. This layer does not change the size of the image, that is, the size of the input image is equal to that of the output image. The output of the layer is as follows:

(1) $$O=\frac{(I-K)+2\times P}{S}+1$$

where, *O* represents the output, *I* represents the input, K is the size of the convolution core, P is the number of extended matrices and S is stride.

(2) ReLU layers: ReLU is an activation function, which does not change the size of the image. The purpose of using ReLU function is to introduce non-linearity, make the outputs of some neurons be zero, reduce the interdependence between parameters, and alleviate the occurrence of over-fitting.

(3) Pooling layers: the function of this layer is to reduce the spatial size of the image and reduce the size of the output image to one-half of the input image.

##### Part II: Regional Proposal Networks (RPN)

Regional proposal networks (RPN) are used to judge that the anchors are foreground or background by softmax, and generate the region proposals. The detailed process of RPN is shown in Figure 2b. The specific steps of RPN are given as follows.

Step 1: after convolution through a 3 × 3 sliding window, VGG16 will output a 512 × 90 × 68 feature map. Every pixel has a 512-dimensional feature vector on the feature map of 90 × 68. Then, the dimension is reduced by 1 × 1 full convolution to accelerate the speed of operation and reduce the calculation parameters.

Step 2: the feature diagram contains two full connection layers. The first full connection layer outputs 18 values and contains 9 anchor boxes with two values each. The two values represent the probabilities of including and not including the object. The second fully connected layer outputs 36 values and also contains 9 anchor boxes with four values each. The four values represent the width, height of ground truth (obtained by image marking) and the predicted values of x and y coordinate, respectively.

Step 3: this step predicts the anchor box containing the object and uses four position regression values to translate and zoom the box. This step will generate a large number of candidate boxes, i.e., the bounding box regression.

Step 4: in this step, the offset is calculated between the anchor box and the ground truth as follows:

(2) $$\left\{ \begin{array}{l} \Delta x=(x*-x_{a})/w_{a}\\ \Delta y=(y*-y_{a})/h_{a}\\ \Delta w=\log(w*/w_{a})\\ \Delta h=\log(h*/h_{a}) \end{array}\right.$$

where, x* and y* represent the center coordinates of the ground-truth box; w* and h* represent the width and height of the ground-truth box, respectively. $x_{a}$ and $y_{a}$ represent the center coordinates of anchor box; $w_{a}$ and $h_{a}$ represent the width and height of anchor box, respectively; Δx and Δy represent the offsets of the center coordinates between the ground-truth box and anchor box, respectively; Δw and Δh represent the offsets of the width and height between the ground-truth box and anchor box, respectively.

##### Part III: RoI Pooling (Region of Interest Pooling)

This layer collects the feature maps of the first part and the proposals of the second part. After the information is synthesized, the feature maps are extracted and sent to the subsequent full connection layers to determine the object category. The function of this part is to reduce the dimension of data and avoid the overfitting. This part outputs the probability of the final recognition accuracy of the fish and a final bounding box.

#### 2.3.2. Location for Fish

The above given position coordinates are directly used to position the fish in the original image. In the three experiments, all images are 1440 × 1080 from the camera and every pixel represents 0.28 mm × 0.37 mm in the image. Faster R-CNN is used to generate proposal boxes in the feature map and map the positions of proposal boxes to the positions of the original images according to the scaling ratio. The center point of the bounding box in the original image is regarded as the point of the position coordinate of the fish, and the document on the location coordinate is further generated in txt format.

The process and output of the used software are as follows:

(1) Firstly, the MATLAB program is used to frame the video to get the data set of fish. The outputs are images in this step.
(2) Secondly, the data set is input into Python for training and output the accuracy of fish recognition and location information.
(3) Finally, the location information is processed to obtain and output the behavior of the fish trajectory map by the MATLAB program.

## 3. Experiment Results

### 3.1. Comparison of Different Object Recognition Method

**Figure 3:**
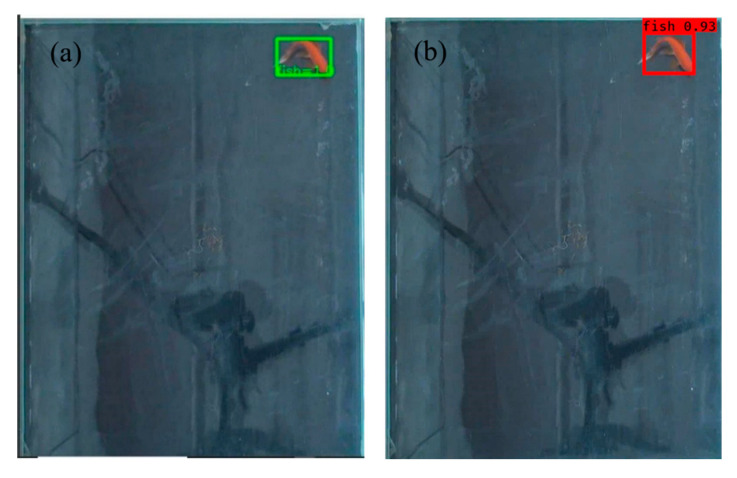
Recognition results of fish. (**a**) recognition by Faster R-CNN, (**b**) recognition by YOLO-V3.

The paper used Faster R-CNN [20] and YOLO-V3 [21] to detect the fish of the same images, respectively. The results of the recognition are shown in Figure 3. The detected fish is represented by a box, and the probability value to be a fish is given along with the box. Figure 3a,b shows the recognition results based on Faster R-CNN and YOLO-V3, respectively.

The statistics of the loss points is shown in Table 1. The average success rate of recognition is 98.13% based on Faster R-CNN, and is 2.47% more than that based on YOLO-V3. It can be seen from the data that Faster R-CNN is more optimal in the recognition accuracy, so the trajectories of fish are plot by the recognition results from Faster R-CNN. From Table 1, it can also be seen that the loss points account for 1.87% of the total, so they do not affect the plotting of the behavior trajectory of fish.

### 3.2. Plot and Analysis of Behavior Trajectory

**Figure 4:**
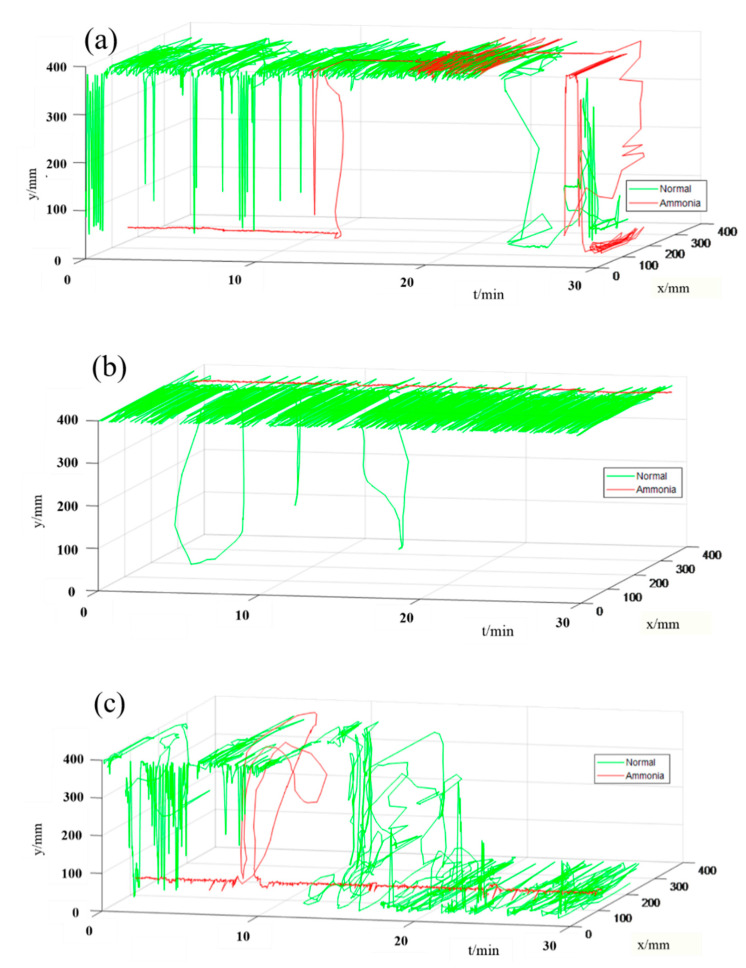
Trajectories of fish in three experiments. (**a**) Fish’s trajectory in the first experiment, (**b**) Fish’s trajectory in the second experiment, (**c**) Fish’s trajectory in the third experiment. The green curve represents the behavior trajectories of the fish in the normal environment, and the red curve represents the behavior trajectories of the fish in the different ammonia environment.

Three experiments were done, and every experiment lasted two days. In three experiments, no any substance was added to the water (i.e., the normal environment) on the first day, and the 0.56 g, 1.12 g and 2.24 g of ammonium chloride was added to the water (i.e., the abnormal environment) in the next day, respectively. In order to make the trajectory clearer to be seen, take only a half-hour trajectory of fish for every experiment. The behavior trajectory of fish is shown in Figure 4. The green curve represents the behavior trajectory of the fish in the normal environment, and the red curve represents the behavior trajectory of the fish in the ammonia environment. As can be seen from the Figure 4, the movement of the fish significantly reduced in the environment with the ammonia compared with the normal environment. For example, the trajectory in the first ten minutes show that the fish was basically stationary in the water in Figure 4a; the fish remained almost motionless for the whole time in Figure 4b; the fish only moved for two minutes and remained motionless in the rest time in Figure 4c. Therefore, we can conclude that fish move much less in ammonium chloride than that in a normal environment.

From Figure 4, it can be seen that the movement of fish decreases, and the fish basically keeps stationary longer in the ammonia environment comparison with that in the normal environment. Table 2 shows the parameters of the experimental environment in the above three experiments.

**Figure 5:**
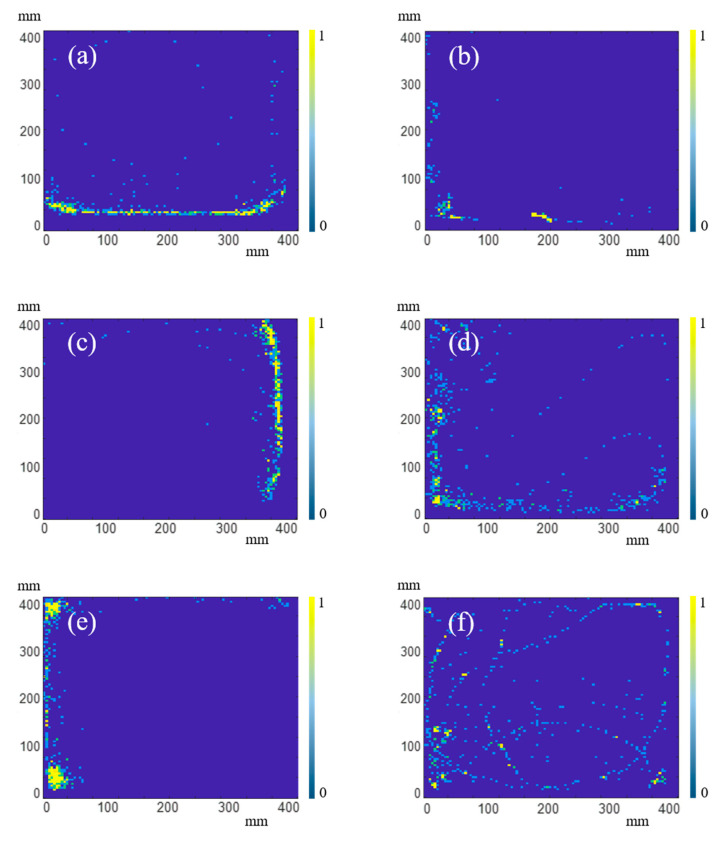
Thermodynamic chart of fish trajectory. (**a**,**b**) are under normal environment and 100 mg/L ammonium chloride environment in the first experiment, respectively; (**c**,**d**) are under normal environment and 200 mg/L ammonium chloride environment in the second experiment, respectively; (**e**,**f**) are under normal environment and 400 mg/L ammonium chloride environment in the third experiment, respectively.

In order to observe the movement of fish directly, a thermodynamic chart of fish trajectory is drawn for the last 10 min of every experiment, as shown in Figure 5. Figure 5a,b is the thermodynamic charts of the behavior trajectory of the fish in the first experiment under the normal environment and the 100 mg/L ammonium chloride environment, respectively; (c) and (d) are the thermodynamic charts of the behavior trajectory of the fish in the second experiment under the normal environment and the 200 mg/L ammonium chloride environment, respectively; (e) and (f) are the thermodynamic charts of the behavior trajectory of the fish in the third experiment under the normal environment and the 400 mg/L ammonium chloride environment, respectively. The color gradient from the blue (corresponding to 0) to the yellow (corresponding to 1) indicates the time change for the stay of fish from short to long, and the yellow points are the positions to stay for the longest time. As can be seen from Figure 5, the movement of fish in ammonium chloride environment is less than that in the normal environment in every experiments.

## 4. Discussion

In the experiments, a monocular camera is used, and the height of water surface is limited for convenience. The images of fish are processed in two-dimensional position coordinates without considering the depth information. However, this does not affect the application of the method under the consideration of depth information, nor does it affect the universality of the proposed approach. That is to say, the proposed approach can use the binocular camera to locate the three-dimensional space coordinates including the depth information, then draw the trajectory of fish and conduct the behavior analysis in three-dimensional space. Of course, the proposed approach in this paper can also be realized by using depth camera.

In the experiments, the ammonia nitrogen detection reagent is selected to measure the concentration of ammonia nitrogen. In fact, the function of ammonia nitrogen detection reagent is to detect the common concentration of NH_3_ and NH_4_^+^. It is worth noting that the high concentration of ammonia nitrogen does not necessarily lead to the poisoning of fish, and the low concentration of ammonia nitrogen may lead to the poisoning of fish because only the NH_3_ is noxious to fish [22]. Whereas the concentration of ammonium chloride and the concentration of NH_3_ have the same trend, so it does not affect the experimental results by using the ammonium chloride to simulate the ammonia environment.

In order to make the trajectory clear and facilitate the observation and analysis of fish behavior, the trajectory of half an hour is taken from the whole behavior trajectory and shown in every experiment. However, this does not affect the analysis of the experimental results, because the trajectory of the selected part is consistent with the phenomena reflected in the whole experimental process. The results show that the fish become inactive when the ammonium chloride is put in the water and the fish often remain motionless. Under the condition of 600 mg/L ammonium chloride, the fish died within 9 h. Therefore, in the actual aquaculture environment, we can conclude that there may exist problems related to the aquaculture environment when the fish is not active, according to the behavior trajectory. Furthermore, we may detect the ammonia concentration in the water to judge whether it is caused by the high ammoniacal concentration or the infected disease and so on, so as to avoid a large number of fish death and the economic loss.

The proposed recognition methods in this paper can detect and recognize the fish more accurately than the traditional methods, but there are still very few failed images to detect fish. The reason may have two aspects: (1) fish is not rigid but is flexible, so the used training date did not include all postures of the fish. (2) the inverted reflection of the fish in the water may affect the recognition results. The few failed recognition of fish does not affect the analysis of experimental results, nor does it affect the plotting of trajectories and further real application.

## 5. Conclusions

In this paper, a deep learning approach is proposed to analyze the behavior of fish, which are different from the existing methods of manual observation and observation by marking objects. The proposed approach provides a new idea and approach for fish behavior research. Through the comparison between Faster R-CNN and YOLO-V3, it is found that the former has better recognition effect. The influence of different concentration of ammonia on the behavior of fish is researched by the deep learning approach. Through this approach, the three-dimensional trajectory of fish can be drawn, and the behavior of fish can be further analyzed. The behavior of fish can further reflect the changes of aquaculture environment or the influence of diseases and so on. This proposed approach is simple, effective and non-invasive, and does not affect the living of fish. This approach not only provides a new approach to research for the behavior of fish, but also provides an effective and feasible approach for monitoring the aquaculture environment of seafood. This proposed approach can also be directly applied to the research or monitoring of other marine organisms, especially in those situations that are not convenient for direct monitoring.

## Figures and Tables

**Table 1:** Statistics of the missed points.

Number of Experiments	Condition of Environment	Method	Lost Points	Total Points	Proportion of Lost Points
First experiment	Normal	Faster R- CNN	16	37,842	0.04%
		YOLO-V3	2321	37,842	6.13%
	100 mg/L Ammonium Chloride	Faster R- CNN	1075	37,931	2.83%
		YOLO-V3	2059	37,931	5.43%
Second experiment	Normal	Faster R- CNN	26	36,767	0.07%
		YOLO-V3	2519	36,767	6.85%
	200 mg/L Ammonium Chloride	Faster R- CNN	578	36,065	1.6%
		YOLO-V3	2346	36,065	6.50%
Third experiment	Normal	Faster R- CNN	176	36,022	0.49%
		YOLO-V3	69	36,022	0.19%
	400 mg/L Ammonium Chloride	Faster R- CNN	2251	36,302	6.2%
		YOLO-V3	349	36,302	0.96%

**Table 2:** Concentration of ammonium chloride and pH value of water under different experiments.

Parameters	Detection Time	First Experiment		Second Experiment		Third Experiment	
		Normal	100 mg/L Ammonium Chloride	Normal	200 mg/L Ammonium Chloride	Normal	400 mg/L Ammonium Chloride
pH value	8:25	7.6	8.0	7.6	8.4	7.6	8.0
	18:35	8.0	8.0	8.0	8.0	8.0	8.0
Concentration of ammonia nitrogen (mg/L)	8:25	0.1	1.8	0.1	1.8	0.1	1.8
	18:35	0.1	1.8	0.1	1.8	0.1	1.8
